# Chaperones Are Necessary for the Expression of Catalytically Active Potato Apyrases in Prokaryotic Cells

**DOI:** 10.1007/s12010-014-0858-6

**Published:** 2014-05-07

**Authors:** Dorota Porowińska, Joanna Czarnecka, Michał Komoszyński

**Affiliations:** Biochemistry Department, Faculty of Biology and Environment Protection, Nicolaus Copernicus University in Torun, Torun, Poland

**Keywords:** NTPDase, Apyrase, Nucleoside triphosphate diphosphohydrolases, Prokaryotic expression system, *Escherichia coli*, Chaperone

## Abstract

NTPDases (nucleoside triphosphate diphosphohydrolases) (also called in plants apyrases) hydrolyze nucleoside 5′-tri- and/or diphosphate bonds producing nucleosides di or monophosphate and inorganic phosphate. For years, studies have been carried out to use both plant and animal enzymes for medicine. Therefore, there is a need to develop an efficient method for the quick production of large amounts of homogeneous proteins with high catalytic activity. Expression of proteins in prokaryotic cells is the most common way for the protein production. The aim of our study was to develop a method of expression of potato apyrase (StAPY4, 5, and 6) genes in bacterial cells under conditions that allowed the production of catalytically active form of these enzymes. Apyrase 4 and 6 were overexpressed in BL21-CodonPlus (DE3) bacteria strain but they were accumulated in inclusion bodies, regardless of the culture conditions and induction method. Co-expression of potato apyrases with molecular chaperones allowed the expression of catalytically active apyrase 5. However, its high nucleotidase activity could be toxic for bacteria and is therefore synthesized in small amounts in cells. Our studies show that each protein requires other conditions for maturation and even small differences in amino acid sequence can essentially affect protein folding regardless of presence of chaperones.

## Introduction

NTPDases (*nucleoside triphosphate diphosphohydrolases*), which in plants are also called apyrases, are enzymes with similar structure and catalytic mechanism. NTPDases are present in all tissues and organs of all plants and animals analyzed so far. These enzymes hydrolyze nucleoside 5′-tri- and/or diphosphate bonds producing nucleosides di or monophosphate and inorganic phosphate [1–5].

NTPDases are commercially used in nucleic acids sequencing, adenylate cyclase activity assay, and blood conservation [6]. For years, studies have been carried out to use both plant and animal enzymes for medicine. Apyrase (ATP diphosphohydrolase) from *Aedes albopictus* hydrolyze the phosphodiester bonds of nucleoside tri and diphosphates to orthophosphate and mononucleotides and thus have potential to attenuate the thrombus [7], whereas engineered and secreted calcium-activated nucleotidase (SCAN) form of human enzyme has been found efficacious in an in vivo model of thrombosis [8], but such studies are limited for want of high-quality purified proteins. Additionally, apyrase isolated from potato is the only commercially available NTPDase. Therefore, there is a need to develop an efficient method for the quick production of large amounts of homogeneous proteins with high catalytic activity.

Expression of proteins in prokaryotic cells is the most common way for the protein production for structural studies (crystallization), catalytic activity analysis, and medicinal and industrial application. However, production of heterologous proteins in bacteria cells often leads to formation of inclusion bodies due to incorrect folding. Insufficiency of folding mechanisms in *Escherichia coli* cells often results from unfavorable small quantity of chaperones compared to very high heterologous protein synthesis. We hypothesized that the provisioning additional copies of different heat shock proteins in bacteria should increase the efficiency of expressed apyrase correct folding and consequent production of catalytically active enzymes.

Therefore, the aim of this work was the expression of three potato apyrase genes in the bacteria *E. coli* in presence and absence of additional copies of GroELS and DnaKJ chaperones sets and to choose the best prokaryotic system and culture condition for their production.

## Materials and Methods

### Materials

#### Sequence of Potato Apyrases

Complementary DNA (cDNA) coding potato apyrases was identified and isolated from cDNA library of Saturna potato tubers. Sequences were deprived of UTRs, signal peptide, and transmembrane domain coding fragments. Modified apyrases sequences were synthesized by GeneScript Corporation and cloned into pET-32a vector with thioredoxin tag.

Library construction as well as identification and isolation of apyrases cDNA was performed in the Biochemistry Department of Nicolaus Copernicus University, Torun, Poland.

### Methods

#### Bacteria Transformation

Transformation of *E. coli* BL21-CodonPlus (DE3)-RIL and BL21 (DE3) strains was performed using heat-shock method. Frozen at −80 °C cells (50 μL) were thawed on ice for 20 min. Then, 1 μl of plasmid (40 ng/μL) was added, gently mixed, and incubated for 20 min in 0 °C. Next, bacteria were placed at 42 °C for 35 s and cooled on ice. Of SOC medium, 250 μL was added, and bacteria were incubated for 1 h at 37 °C with gentle shaking. Transformed cells grew overnight on LB plates supplemented with glucose (0.5 %) and ampicillin (50 μg/mL).

Transformation of ArcticExpress (DE3)RIL was performed using heat-shock method with mercaptoethanol addition according to manufacturer’s protocol.

#### Overexpression of Potato Apyrases (StAPY4, StAPY 5, StAPY 6) in *E. coli* BL21-CodonPlus (DE3)-RIL Strain

Single colony of transformed bacteria was transferred to liquid Lysogeny broth medium (LB) supplemented with glucose (0.5 %), ampicillin (50 μg/mL), and chloramphenicol (34 μg/mL). Bacteria were incubated overnight in 37 °C with vigorous shaking. The next day, cells were transferred to fresh LB medium and cultured with shaking in 37 or 22 °C. When culture OD_600_ reached 0.5, bacteria were induced to apyrase expression with 1 mM isopropyl B-D-1-thiogalactopyranoside (IPTG) and incubated under these conditions for 3 h. After that, bacteria were cooled on ice and centrifuged at 10,000×*g* for 20 min. Bacteria pellet was suspended in 25 mM Tris-HCl buffer, pH 7.0.

Autoinduction method was performed in ZYM medium supplemented with ampicillin (50 μg/mL) and chloramphenicol (34 μg/mL) using Studier method [9]. Bacteria cells were cultured in 20 °C for 24 h with vigorous shaking. Then, bacteria were cooled on ice and centrifuged at 10,000×*g* for 20 min. Bacterial pellet was suspended in 25 mM Tris-HCl buffer, pH 7.0, and stored in −20 °C.

#### Overexpression of Potato Apyrases (StAPY4, StAPY 5, StAPY 6) in *E. coli* ArcticExpress (DE3)RIL Strain

Overexpression of potato apyrases in *E. coli* ArcticExpress (DE3)RIL strain was performed according to manufacturer’s protocol. After apyrase expression bacteria were cultured for 24, 48, and 72 h. Then, bacteria were cooled on ice and centrifuged at 10,000×*g* for 20 min. Bacteria pellet was suspended in 25 mM Tris-HCl buffer, pH 7.0, and stored at −20 °C.

#### Co-expression of Potato Apyrases (StAPY4, 5, 6) in *E. coli* BL21 (DE3) Strain

Single colony of transformed bacteria cells was transferred to liquid LB medium with addition of glucose (0.5 %), ampicillin (50 μg/mL), and chloramphenicol (20 μg/mL) and incubated overnight at 37 °C with vigorous shaking. The next day, cells were transferred to fresh medium with the same supplements and cultured under similar conditions.

##### One-Step Cultivation

When OD_600_ of culture reached 0.4, bacteria were induced to chaperones synthesis with the use of l-arabinose (4 mg/mL) and tetracycline (10 ng/mL) and the temperature of cultivation was lowered to 25 °C. Bacteria were cultured under these conditions till optical density achieved 0.8. Then, IPTG was added to final concentration 1 mM for apyrase synthesis induction and cells were incubated for 2 h under the same conditions. After that, cells were cooled on ice and centrifuged at 10,000×*g* for 20 min. Bacteria pellet was suspended in 25 mM Tris-HCl buffer, pH 7.0, and stored at −20 °C [10].

##### Two-Step Cultivation

When OD_600_ of culture reached 0.4, bacteria were induced to chaperones synthesis with the use of l-arabinose (4 mg/mL) and tetracycline (10 ng/mL) and the temperature of cultivation was lowered to 25 °C. At optical density equal 0.8, bacteria were cooled on ice and centrifuged at 5,000×*g* for 20 min. Cell pellet was suspended in the same amount of liquid LB medium with supplements, and 1 mM IPTG was added for apyrase synthesis induction. Bacteria were cultured overnight in 25 °C with shaking. The next day, cultures were cooled on ice and centrifuged at 10,000×*g* for 20 min. Bacteria pellet was suspended in 25 mM Tris-HCl buffer, pH 7.0, and stored at −20 °C.

#### Analysis of Nucleotidase Activity

Catalytic activity of potato apyrase was performed using Fiske-Subbarow method following modification by Komoszyński and Skalska [11].

### Analysis of Nucleotidase Activity on Polyacrylamide Gel

After electrophoresis under non-denaturing conditions, gel was placed in 60 mM HEPES buffer containing 2 mM ATP or ADP and 10 mM CaCl_2_ and incubated at 37 °C till the white precipitates of calcium phosphate was noticed.

### Analysis of Potato Apyrase 5 in *E. coli* Cells

#### Isolation of Total RNA and Reverse Transcription

Isolation of total RNA from bacteria cells and reverse transcription reaction was performed using TRI Reagent (Sigma-Aldrich) and First Strand cDNA Synthesis Kit (Fermentas) respectively, according to manufacturer’s protocols.

#### PCR Reaction

PCR reaction was performed on cDNA obtained in reverse transcription reaction using PCR Master Mix 2x reagent (Fermentas) and two specific primers for apyrase: 5′-GATCTTGGTGGTGGTTCAGTCC-3′ and 5′-CTTTAGCTGCATTTAAGTATTGAATTGG-3′ [12]. As a positive reaction, pET32a plasmid carrying StAPY5 sequence was used. PCR was conducted according to the following program: initial matrix denaturation 3 min, 95 °C; PCR reaction (35 cycles) (a) matrix denaturation 60 s, 94 °C, (b) primers hybridization 60 s, 51 °C, and (c) elongation 2 min, 15 s, 72 °C; and final elongation 5 min, 72 °C [12].

#### Electrophoresis in Polyacrylamide Gel

Both denaturing and non-denaturing electrophoresis in polyacrylamide gel were performed using Ogita and Markert method [13]. For protein separation by sodium dodecyl sulfate polyacrylamide gel electrophoresis (SDS-PAGE), 4 % stacking gel and 10 % running gel were used. For non-denaturing-PAGE, 3 % stacking gel and 5 % running gel containing 1 % Triton X-100 and 5 % ethylene glycol were used.

### Immunoprecipitation

Immunoprecipitation was performer using Bollag and Edelstein method [14]. For reaction, rabbit polyclonal anti-thioredoxin antibody was used. Of protein solutions, 100 μL (1 mg of protein) was mixed with 5 μL of antibodies (10 mg/mL) and incubated on ice for 2 h with shaking. In the next step, 100 μL of acrylic beads with immobilized A protein was added and incubated for 1 h under the same conditions. Beads were washed twice with 25 mM Tris-HCl buffer, pH 7.0, and incubated with 50 μL of 25 mM glycine-HCl buffer, pH 2.5, for 5 min. Next, beads were centrifuged and the supernatant was neutralized with 25 mM Tris buffer.

## Results and Discussion

The aim of study was to develop a method of expression of potato apyrase (StAPY) genes in bacterial cells under conditions that allowed the production of catalytically active form of these enzymes and would facilitate the analysis of their physic-chemical properties.

As a rule, attempts at purification of those enzymes from their natural plant and animal sources have been ineffective. Isolation of proteins is multistage and is often expensive and inefficient in producing high purity products [15–17]. Moreover, animal NTPDases are sensitive to detergents used in purification processes, which substantially reduces their activity [18]. Furthermore, commercially available potato apyrases are not homogenous since they show additional activity of alkaline phosphatase, acid phosphatase [19], and 5′-nucleotidase (personal studies).

The best way to produce substantial amounts of recombinant proteins is their synthesis in prokaryotic expression systems. However, so far, expression of plant and animal recombinant apyrases in *E. coli* cells has almost always resulted in formation of inclusion bodies (IB) [20–24], with an only exception of *Mimosa pudica* where soluble and catalytically active apyrase enzyme synthesis has been demonstrated [17, 25]. The reason for the formation of inclusion bodies is usually a toxic effect of synthesized proteins on bacterial cells or incorrect folding of overexpressed polypeptides resulting from limitations of prokaryotic cells in eukaryotic protein synthesis (errors in the synthesis of a polypeptide chain, reducing environmental bacterial cytosol) and/or very high level expression of heterologous proteins (inefficient intracellular mechanisms of polypeptides folding) [26].

The choice of appropriate bacteria strain for protein overproduction is the key for its proper and efficient synthesis. So far, both plant and animal NTPDases were expressed in *E. coli* BL21 (DE3) strain [20–24, 27] but with attenuated efficacy because of formation of IB. For the expression of StAPY4, 5, and 6, we chose BL21-CodonPlus (DE3) and ArcticExpress (DE3)RIL strains, which are better adjusted for eukaryotic protein expression.

**Fig. 1:**
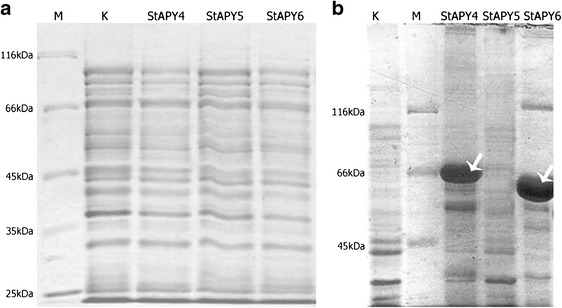
SDS-PAGE of *E. coli* BL21-CodonPlus (DE3)-RIL proteins. **a** Soluble protein fractions (supernatant). **b** Insoluble protein fractions (pellet). *M* molecular mass marker, *K* control culture, *StAPY4* culture induced to apyrase 4 synthesis, *StAPY5* culture induced to apyrase 5 synthesis, and *StAPY6* culture induced to apyrase 6 synthesis. *White arrows* overproduced proteins of StAPY4 and StAPY6, respectively. Figure presents protein separations observed in all analyzed culture variants (see “Materials and Methods”)

**Fig. 2:**
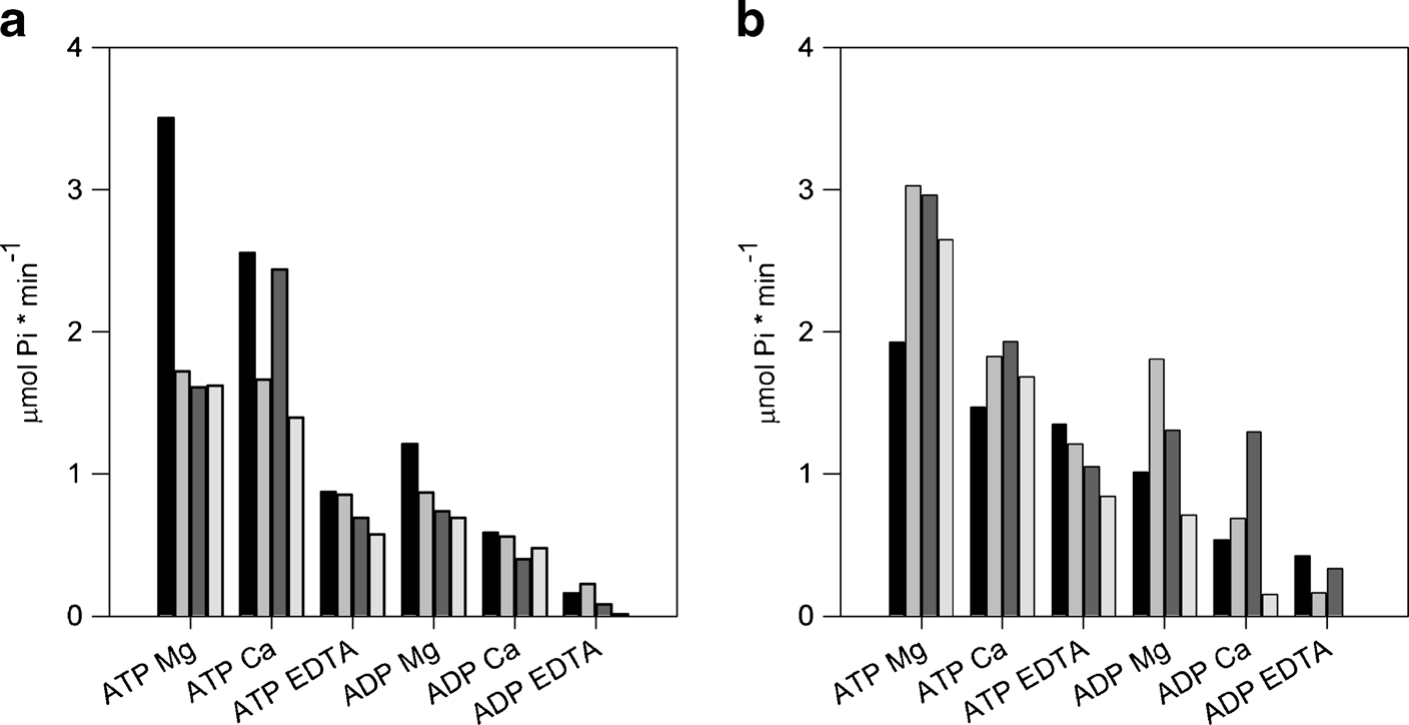
Nucleotidase activity in *E. coli* BL21-CodonPlus (DE3)-RIL in autoinduction method. **a** Total activity in supernatant. **b** Total activity in pellet. *Black* control culture, *gray* culture induced to apyrase 4 synthesis, *dark gray* culture induced to apyrase 5 synthesis, and *light gray* culture induced to apyrase 6 synthesis

Expression of potato apyrases (*Solanum tuberosum apyrase*, i.e., *StAPY*) in *E. coli* BL21-CodonPlus (DE3) strain resulted in overexpression of two of three analyzed apyrases, namely, StAPY4 and StAPY6, but synthesized proteins formed insoluble and inactive inclusion bodies (Fig. 1). Even change of culture conditions such as lower culture temperature of 22 °C in combination with IPTG induction did not mitigate the formation of IB. Of note, *E. coli* induction by IPTG also resulted in inclusion bodies formation in the case of synthesis of human NTPDase5 [21] and 6 [20], ectodomain of rat NTPDase1, 2, and 3 [24], AtAPY1 and AtAPY2 from *Arabidopsis thaliana* [22], GS52 from soybean [23], and MP67 from *Mimosa* [17]. The autoinduction method [9, 28], in turn, which combines low culture temperature (20 °C) and gradual induction of bacteria, still leaded to the formation of inclusion bodies. This is not consistent with the results obtained by Vivian et al. [27]. However, in this method a small increase of ATP and ADP hydrolysis in pellet in the presence of Mg^2+^ and Ca^2+^ ions was observed (Fig. 2). This suggested that at least some of the apyrase accumulated in inclusion bodies enzymes might have had correct conformation.

**Fig. 3:**
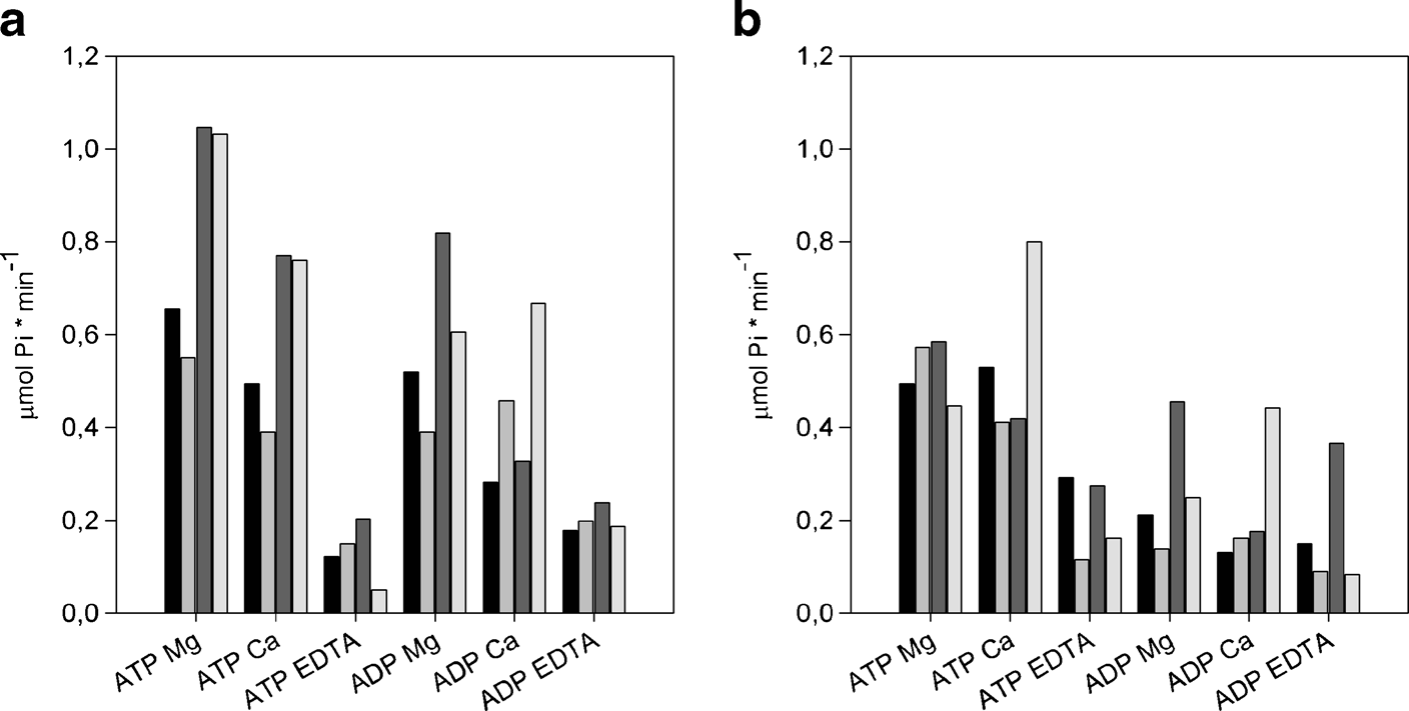
Nucleotidase activity in *E. coli* BL21 ArcticExpress (DE3)RIL, 72 h after bacteria induction to apyrase synthesis. **a** Total activity in supernatant. **b** Total activity in pellet. *Black* control culture, *gray* culture induced to apyrase 4 synthesis, *dark gray* culture induced to apyrase 5 synthesis, and *light gray* culture induced to apyrase 6 synthesis

Greater reduction of culture temperature (to 13 °C) in ArcticExpress (DE3)RIL strain, in turn, resulted in lost of overproduction of all analyzed potato apyrases. However, in this bacteria strain, we observed an increase of nucleotidase activity in supernatant at 72 h of bacterial induction for StAPY5 and StAPY6 synthesis compared to control cultures. Hydrolysis of ATP and ADP by both proteins was stimulated with divalent cations (Mg^2+^ ≥ Ca^2+^) and inhibited by EDTA (Fig. 3).

### Co-expression of StAPY4, StAPY5, and StAPY6 with Bacterial GroELS and DnaKJ Chaperones

**Fig. 4:**
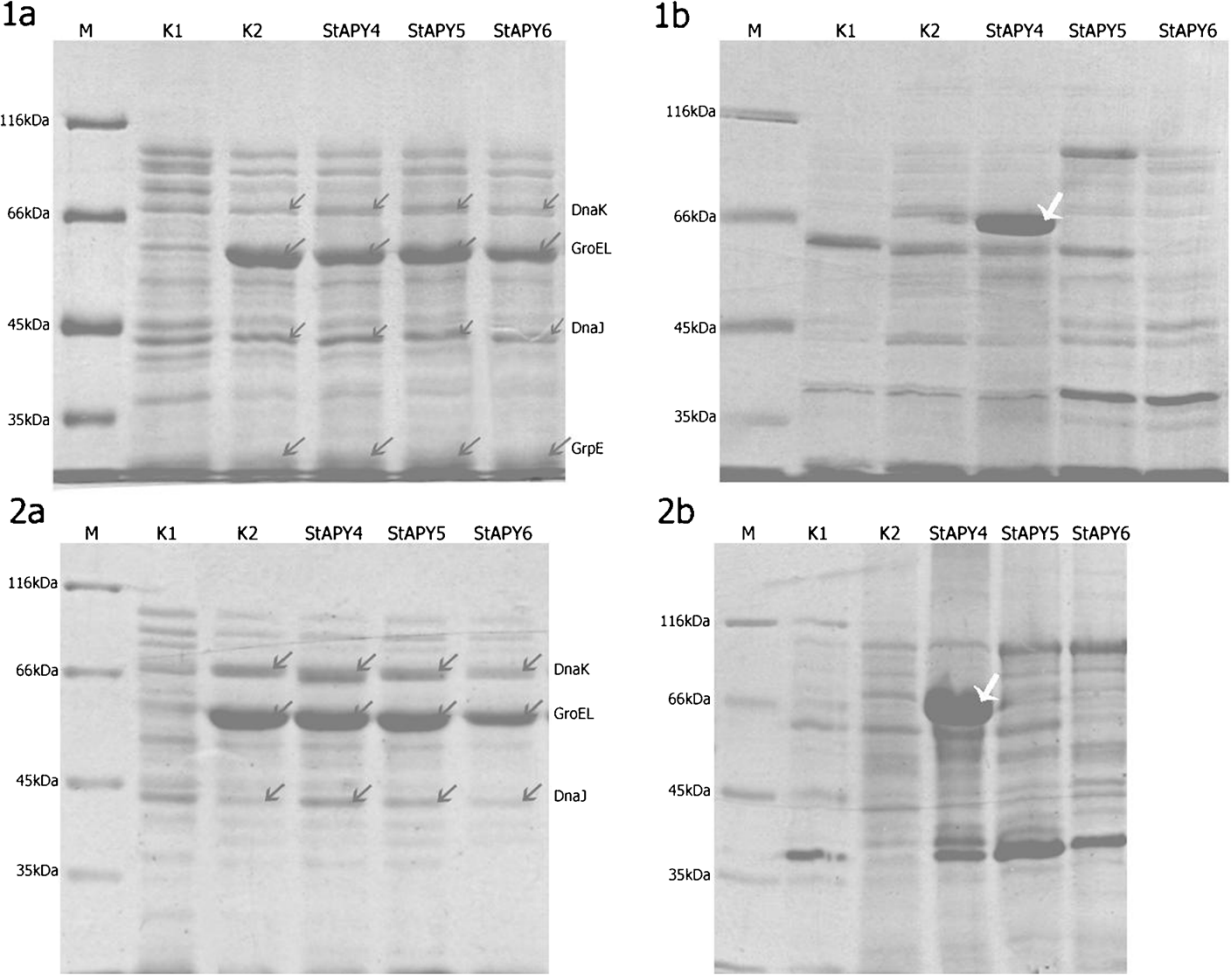
SDS-PAGE of bacteria *E. coli* BL21 (DE3) proteins after co-expression of potato apyrases with chaperones. *1* one-step cultivation and *2* two-step cultivation. **a** Supernatant. **b** Pellet. *M* molecular mass marker, *K1* control culture, *K2* control culture with induction of chaperone expression only, *StAPY4* culture induced to apyrase 4 synthesis, *StAPY5* culture induced to apyrase 5 synthesis, and *StAPY6* culture induced to apyrase 6 synthesis. *Black arrows* overproduced chaperones, *white arrows* overproduced protein of StAPY4

Reducing the expression of recombinant apyrase in bacteria did not limit the formation of inclusion bodies. Thus, in our study, we decided to co-express StAPY4, 5, and 6 with heat shock proteins which have been demonstrated as chaperones for correct folding of the nascent proteins [29]. For our studies, we chose GroEL, GroES, DnaK, and DnaJ heat shock proteins, which are naturally present in all *E. coli* cells [30, 31]. Co-expression of above chaperones with analyzed potato apyrases was performed in two culture variants (see “Materials and Methods”). In the presence of chaperones, potato apyrase 4 was overexpressed in bacteria but it formed inclusion bodies (in both one- and two-step cultivation). Potato apyrase 6 was inefficiently synthesized in BL21-CodonPlus (DE3)-RIL strain and did not co-express with chaperones (Fig. 4). The lack of co-expression of this enzyme and chaperones in our cultures is difficult to explain. Nevertheless, we think that GroEL and DnaK are quite large proteins with the molecular masses of 60 and 70 kDa, respectively [29]. As such, their biosynthesis will require a lot of energy and overexpression of these chaperones could charge the bacteria metabolism at the cost of synthesis of apyrases, with StAPY4 being an exception where we did not encounter such a problem. Potato apyrase 5 did not co-express with chaperones (Fig. 4).

Co-expression of heterologous proteins and molecular chaperones may be chaperone specific [10]. Therefore, we checked co-expression effect of different sets of chaperones (DnaKJ and GroELS systems) on potato apyrases. Our results from each variant reflected rather opposite trends (data not show). This led us to conclude that correct three-dimensional structure of potato apyrase did not depend on co-expressed chaperone set.

**Fig. 5:**
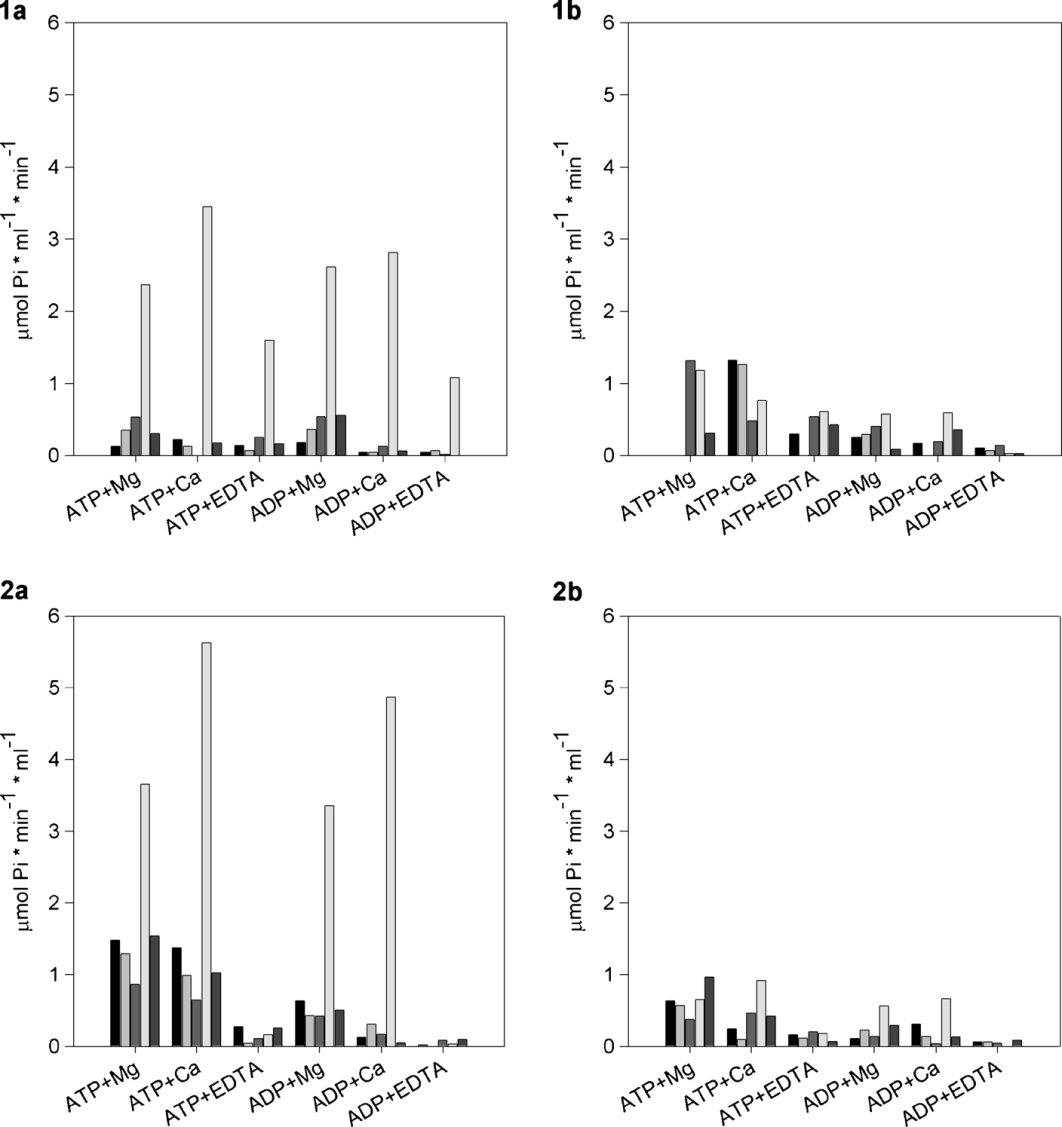
Nucleotidase activity in *E. coli* BL21 (DE3) after potato apyrases and chaperones co-expression. *1* one-step cultivation and *2* two-step cultivation. **a** Total activity in supernatant. **b** Total activity in pellet. *Black* control culture, *gray* control culture with induction of chaperone expression only, *dark gray* culture with co-expression of chaperones and apyrase 4, *light gray* culture with co-expression of chaperones and apyrase 5, and *darker gray* culture with co-expression of chaperones and apyrase 6

Despite lack of overexpression, in supernatants, after the co-expression of chaperones and StAPY5 (in both one- and two-step culture variant), we observed a distinct increase in ATP and ADP hydrolysis in comparison to control cultures (Fig. 5). Hydrolysis of those nucleotides was activated by divalent metal ions (Ca^2+^ > Mg^2+^), and the ratio of ATP/ADP degradation was near 1. This suggested that apyrase 5 was expressed in bacteria in correct active form (StAPY5). However, such a high nucleotide hydrolyzing activity was probably toxic for bacteria and that is why we observed the production of this protein only in small amounts. Elimination of chaperone’s inductors in two-step cultivation before apyrase expression induction resulted in much higher ATP and ADP hydrolytic activity. Thus, this culture variant was more efficient (near 1.5 times) for StAPY5 than one-step cultivation although it was time consuming.

### Identification of Potato Apyrase 5 in *E. coli* Cells

**Fig. 6:**
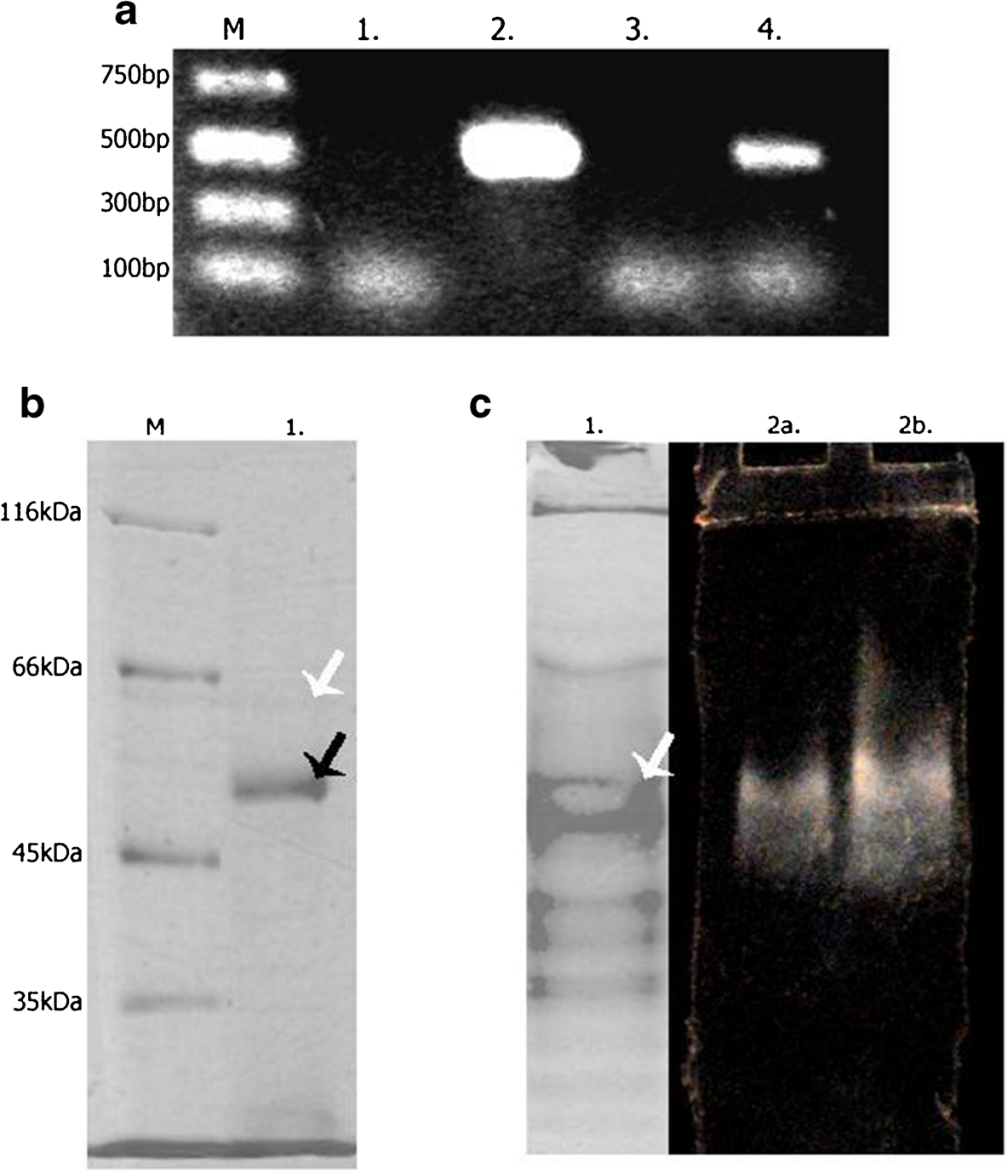
Identification of potato apyrase 5 in *E. coli* BL21 (DE3) cells. **a** PCR method. *M* marker, *1* negative control, *2* positive control, *3* PCR product on mRNA matrix isolated from bacteria cells induced to chaperones expression only, *4* PCR product on mRNA matrix isolated from bacteria cells induced to chaperones and StAPY5 expression. **b** Immunoprecipitation. *M* molecular mass marker and *1* proteins after immunoprecipitation. *White arrow* potato apyrase 5 (∼65 kDa) and *black arrow* heavy chain of antibody used in experiment. Thioredoxin was localized on N-terminus of apyrase 5 as a fusion tag. Therefore, proteins with a molecular mass lower than 65 kDa visible on electrophorogram are the fragments of potato apyrase 5 chain. **c** Electrophoresis in non-denaturing conditions of proteins in bacteria supernatant after co-expression of potato apyrase 5 with chaperones. *1* proteins after Coomassie Brilliant Blue R-250 staining, *2* nucleotide hydrolysis on gel: *a* ATP as a substrate and *b* ADP as a substrate

Some of chaperones used in our studies might have had an auto-ATPase activity in the presence of divalent magnesium ions [30, 31], implying that observed heightened nucleotidase activity could in fact be ascribed to chaperones activity. However, in control culture in which only chaperones were overexpressed, we did not observe an increase in nucleotide hydrolysis (Fig. 5). Furthermore, observed activity was dependent on calcium ions rather than magnesium in both ATP and ADP degradation. Besides, transcript analysis and immunoprecipitation in the presence of specific antibody finally confirmed the presence of StAPY5 in bacterial cells (Fig. 6a, b). It strongly suggested the co-expression of StAPY5 and chaperones in *E. coli* BL21 (DE3) strain.

Reaction with ATP as well as ADP as a substrates performed on polyacrylamide gel after electrophoresis in non-denaturing conditions confirmed the presence of only one nucleotide hydrolyzing enzyme (one white band of calcium phosphate precipitate) with molecular mass about 60 kDa (Fig. 6c). The band observed after hydrolysis of ADP was much more intense than the one from the reaction with ATP. This implies that recombinant apyrase 5 is rather ADP than ATP specific enzyme. This agrees with the results obtained for activity revealed in supernatant (Fig. 5).

## Conclusions

Our studies on expression of potato apyrases (StAPY4, 5, 6) in bacteria show that the best *E. coli* strain for overexpression of apyrase 4 and 6 is BL21-CodonPlus (DE3)-RIL. Regardless of culture conditions and induction method, we were able to overproduce both the enzymes, but they were always accumulated in inactive inclusion bodies. These results are consistent with the majority of so far carried out studies. Expression of recombinant NTPDases, both plant and animal, in bacterial cells, almost always resulted in the formation of insoluble and inactive aggregates [20–24]. Only in the case of *M. pudica* apyrase, a soluble and catalytically active enzyme was achieved, in addition to IB [17, 25]. Soluble and catalytically active NTPDase also obtained in a prokaryotic system was Lp1NTPDase derived from the bacterium *Legionella pneumophila*. The positive effect of this experiment resulted probably from a bacterial origin of this enzyme [27].

However, in our study, we show for the first time that the co-expression of molecular chaperones and StAPY5 allowed correct folding of this enzyme in vivo. It matched production of catalytically active GS52 apyrase from soybean when co-expressed with cycloamylose an “artificial chaperone” in vitro [23]. Paradoxically, it is not fully clear for us why did we obtain active form of only one apyrase variant (StAPY5) out of three apyrases analyzed under similar conditions. We expected that their expression in bacteria cells would be comparable because of their high (89–97 %) sequence homologies. As a consequence, we are tempted to suggest that even small differences in amino acid sequence can essentially affect protein folding regardless of presence of chaperones. But the results from StAPY4 and StAPY5 are hard to explain especially when the homologies between their amino acid stand at 97 %. Apyrase 4 was produced efficiency in almost all analyzed bacterial strains along with the formation of inclusion bodies. Apyrase 5, however, did not overexpress in any of analyzed bacterial strains. The only way for its production in correct active form in vivo was ascribable to its refolding via molecular chaperones. However, the reason of the lack of apyrase 5 overexpression may be due to its high catalytic activity. Intensive hydrolysis of ATP and ADP (which we observed) could cause the death of bacterial cells, and as a consequence, the amount of enzyme produced was very little. In two-step cultivation, after induction to StAPY5 production, the growth of bacteria slows down (data not shown). It gives credence/validity to our suggestion that apyrase 5 is toxic for bacteria cells.
